# COMMUNITY DISCHARGE AFTER SKILLED NURSING FACILITIES BETWEEN MEDICARE FEE-FOR-SERVICE AND MEDICARE ADVANTAGE

**DOI:** 10.1093/geroni/igae098.0077

**Published:** 2024-12-31

**Authors:** Maricruz Rivera-Hernandez, Lin-Na Chou, Amit Kumar

**Affiliations:** Brown University School of Public Health, Providence, Rhode Island, United States; University of Utah, Salt Lake City, Utah, United States; University of Utah, Salt Lake City, Utah, United States

## Abstract

The purpose of this study was to assess differences in rates of successful community discharge among Medicare Advantage (MA) and fee-for-service (FFS) enrollees by race/ethnicity. We linked the Master Beneficiary Summary File, Medicare Provider and Analysis Review data, and the Minimum Data Set. We identified 2,412,731 Medicare enrollees who were admitted to a skilled nursing facility in 2020. We assessed community discharge within 100 days remaining out of the skilled nursing facility for 30 days (LTC focus; Rivera-Hernandez et al., 2019). Approximately, 34.48% beneficiaries were enrolled in a MA plan at admission. A higher proportion of MA beneficiaries were discharged to the community compared to FFS beneficiaries (53.64% vs. 42.70%). Among MA beneficiaries, 54.50% of Hispanic beneficiaries and 47.51% of Black beneficiaries were discharged to the community compared to 53.31% among white enrollees. However, 45.85% of white FFS beneficiaries were discharged to the community compared to 38.71% Hispanic and 34.60% of Black FFS beneficiaries. After adjusting for clinical, demographic characteristics and facility fixed-effects, rates of discharge to the community remained approximately 4.87 percentage points (p.p.) higher (95% CI, 4.72 to 5.02) among MA compared to FFS enrollees. In addition, this difference among FFS and MA beneficiaries remained wider among Black (3.43 p.p., 95% CI, 3.05 to 3.81) enrollees when compared to the difference among white FFS and MA enrollees. The observed differences in successful discharge to the community among Medicare enrollees suggest the need to promote equity in the transition of care among the underserved adult population.

